# Cardiac ventricular dimensions predict cognitive decline and cerebral blood flow abnormalities in aging men

**DOI:** 10.1186/1471-2318-13-45

**Published:** 2013-05-15

**Authors:** Linda Furuäng, Per Wollmer, Arkadiusz Siennicki-Lantz, Sölve Elmståhl

**Affiliations:** 1 Division of Geriatric Medicine, Department of Health Sciences, Lund University, Skåne University Hospital, Jan Waldenströms gata 35, Malmö SE-205 02, Sweden; 2 Division of Clinical Physiology, Department of Clinical Sciences, Lund University, Skåne University Hospital, Malmö, Sweden

**Keywords:** Aging, Cerebrovascular circulation, Cohort study, Cognition, Hypertension, Left ventricular dimension

## Abstract

**Background:**

The aims of this study are to examine possible associations between left cardiac ventricular measures in sixth decade and cognitive performance, both cross sectionally and longitudinally, and to examine if left cardiac ventricular measures could predict future changes in cerebral blood flow (CBF).

**Methods:**

211 elderly men from a cohort of the population study “Men born in 1914” completed M-mode echocardiography and a cognitive test battery at age 68. The cognitive test battery was repeated at age 81. CBF was estimated with ^99mTc^-HMPAO SPECT in 72 survivors at age 83. Cognitive performance at baseline and at 1^st^ follow up and CBF at 1^st^ follow up were analysed in relation to left ventricular internal dimension in diastole (LVIDd mm/m^2^) and fractional shortening (FS).

**Results:**

Subjects with enlarged LVIDd at age 68 had poorer results on verbal and speed-performance tests at baseline and on verbal and visuo-spatial tests 14 years later on. Low FS was associated with decreased results on visuo-spatial tests at baseline. There was an inverse relationship between LVIDd and both verbal and spatial ability at the baseline and after 14 years of follow-up. Normotensive men with lower FS had also decreased CBF in a majority of brain areas 14 years later.

**Conclusions:**

Mild echocardiographic abnormalities in 68 ys.-old men, as increased LVIDd and lower FS, are associated with lower cognitive test results and may predict cognitive decline and silent cerebral perfusion abnormalities 14 years later.

## Background

Over the years, several studies have reported an association between congestive heart failure and cognitive decline [1,2]. Almeida and Flicker [2] presented in their review several reports on the role of congestive heart failure in a generalized cognitive impairment, concerning memory and attention deficits. However, the number of studies was low and with several limitations concerning sample selection. Left ventricular hypertrophy (LVH) has been reported to be an independent predictor of stroke in essential hypertension [3]. Increasing left ventricular mass predicts a higher incidence of cardiovascular events [4] and is also associated with cognitive decline in the elderly [5,6]. In patients with essential hypertension, LVH was associated with a reduction of regional cerebral blood flow (CBF) in the striatum area [7]. An increase in left ventricular internal dimension (LVID), both end-systolic and end-diastolic (LVIDs and LVIDd), is a risk factor for congestive heart failure in persons who have not had a myocardial infarction [8]. Fractional shortening (FS) is one parameter of assessing left ventricular systolic function. In the study of Lauer et al. 1992, low fractional shortening was associated with an increased risk for new events of cardiovascular disease [9]. Studies on the relationship between FS and cerebral function remain however sparse. Left ventricular ejection fraction (LVEF) is nowadays assessed in order to describe left ventricular function. Low, but also high LVEF have been suggested to be associated with poorer cognitive performance [10]. Further studies on the relationship between cardiovascular risk factors and possible dementia are asked for [11,12].

The aims of this study are to examine, in a cohort of men, possible associations between their left cardiac ventricular measures in sixth decade and cognitive performance, both cross sectionally and longitudinally, and to assess if left cardiac ventricular measures could predict changes in cerebral blood flow in those men who reached age 83.

## Methods

### Population

**Figure 1:**
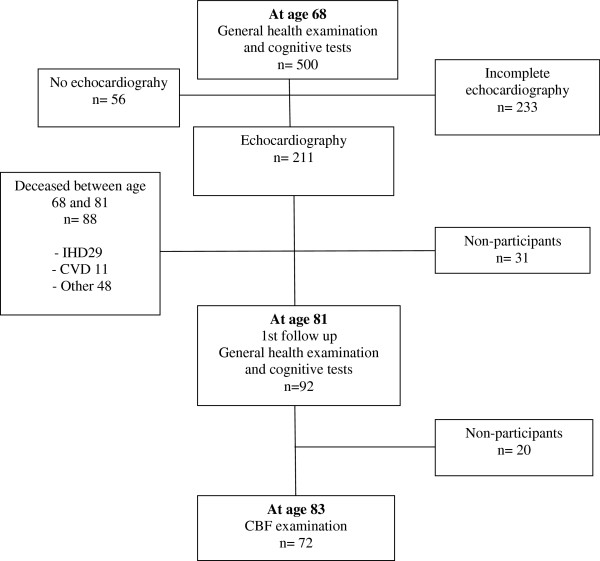
Participation of the cohort “Men born in 1914”.

“Men born in 1914” is a prospective population study which started in 1968. The cohort included all men born even months in 1914 and residing in a city of Malmö, Sweden. 703 (87%) out of 809 men agreed to participate in 1968–69. At age 68, 500 out of 560 (89%) men participated in a general health examination which included cognitive tests (Figure 1). 444 (89%) out of the 500 men, underwent echocardiography. 211 (48%) men had complete echocardiography regarding left cardiac ventricle and were included in the analyses. At age 81, 92 survivors participated in a follow-up including a general health examination and a battery of cognitive tests. The following year, at age 82–83, 72 survivors underwent CBF measurements. Background data were published previously [13,14]. Information on background factors was taken from the examination at the age of 68 years.

### Questionnaire and health examination

The study subjects completed a health and sociodemographic questionnaire at the age of 68 and 81. Smoking habits were categorised into three groups: current smokers (a consumption of at least 1 g of tobacco/day), former smokers (regularly smoking for at least a year) and non-smokers. Alcohol consumption was defined as no (no use of alcohol), rarely (once a week) and regularly (several times a week). Ankle-brachial pressure index (ABPI) was calculated and ABPI < 0.9 was defined as peripheral arterial disease. Diabetes mellitus was defined as a fasting blood glucose level >7 mmol/l or medication for diabetes mellitus [15]. Office blood pressure (BP) was measured sphygmomanometrically with the subject in a sitting position. Hypertension was defined as systolic and diastolic brachial blood pressure >160 mm Hg and >90 mmHg, respectively, or being on antihypertensive medical treatment [16]. Hyperlipidemia was defined as fasting triglycerides ≥ 2.3 mmol/l and cholesterol ≥ 6.5 mmol/l [17,18]. Angina pectoris was defined according to the Rose questionnaire [19]. Stroke was defined according to the ICD-9 classification (430.00-438.99). Education was classified into four groups: (1) 0–7 ys., (2) 7-9ys, (3) 10-13ys. (4) higher education. BMI was calculated as weight (kg)/height (m^2^).

### Echocardiography

M-mode echocardiograms were obtained using a Diasonics machine, with the study subjects lying on the left side. The recordings were assessed by two experienced examiners. Measurements of left ventricular internal dimensions at end-diastole (LVIDd) and at end-systole (LVIDs) were obtained in M-mode according to the standards at the time [20]. We used values corrected for body surface area (BSA), mm/m^2^. Left ventricular dimensions were categorized as follows: normal LVIDd if 22–31 mm/m^2^, mildly abnormal if 32–34 mm/m^2^, moderately abnormal if 35–36 mm/m^2^ and severly abnormal if ≥ 37 mm/m^2^[21,22]. LVIDd ≥ 32 mm/m^2^ was defined as large in our study. Concerning reference values for LVIDs there is a discrepancy in the literature [21,22]. Endocardial fractional shortening (FS) was used as a measure of the contractility of the left cardiac ventricle and calculated according to the formula: ((LVIDd-LVIDs)/ LVIDd) × 100 ( LVIDd and LVIDs not corrected for BSA) [23]. Normal range of FS is 25-43% [21].

### Measures of cognitive functions

At the age of 68 and 81 years, study subjects were examined by one and the same psychologist at both examinations with a battery of cognitive tests: (a) Test of Synonyms, a test on general verbal ability with a maximum score of 30 [24]; (b) Block Design, a spatial ability test [25]; (c) Digit Symbol Substitution test, measuring: psychomotor speed, visual-motor coordination, concentration, sustained attention and cognitive flexibility with a maximum score of 90 [25] and (d) Benton Visual Retention Test, which tests immediate visual and spatial memory with a maximum score of 10 [26]. At follow-up, at age 81, a repeated measurement was made with the same test battery, adding the Mini Mental State Examination (MMSE) with a maximum score of 30 [27].

### Cerebral blood flow measurement (rCBF)

Cerebral blood flow measurement was performed with single photon emission computed tomography (SPECT) using ^99m^ Tc-hexamethylpropyleneamine oxime (^99m^Tc HMPAO; Ceretec®, Amersham Int.), as previously described [13]. Regions of interest (ROIs) were positioned and scaled to the recorded SPECT. The 10 transaxial slices (1 cm thick), based on the external borders of each slice, included following ROIs in each hemisphere: frontal, temporal, parietal, occipital, and basal ganglia. The estimated value in each ROI was expressed as a percentage of the mean cerebellar count density.

### Statistics

The SPSS 20.0 statistical package was used for the analyses. The study group was divided into deceased before follow-up and survivors to follow-up. Differences in background data between the deceased and survivors were examined by using the chi-squared test for categorized variables, Kruskal Wallis test or Fischer’s exact test in case of small frequencies and t-test for quantitative variables. The same statistical methods were used in exploring differences between the participants and excluded subjects. The t-test was used in comparing cognitive performance in subjects with high LVIDd versus subjects with normal LVIDd. A General Linear Model was used to test associations between left ventricular measures (dependent variable) and the results of cognitive tests and cerebral blood flow, adjusted for education and appropriate vascular risk factors. In the latter analysis, the material was stratified for hypertension, being a confounder to both cardiac and cerebral function A *p*-value < 0.05 was considered significant.

### Ethics

The study was approved by the local ethics committee at Lund University (LU 111–82). All subjects gave their informed consent.

## Results

### Background data

Background data for all subjects at age 68, who were examined by echocardiography, and for the subgroups: deceased between age 68 and 81 and survivors to age 81 are presented in Table 1. Among the deceased subjects, there was a higher proportion of peripheral arterial disease, hypertension and angina pectoris compared to the survivors. Cognitive test results at the baseline examination, especially Block Design test, Digit Symbol Substitution test and Benton Visual Retention Test, had significantly lower scores in the deceased group compared to the survivors (Table 1). When examined at age 81, all (94%) but six of the study subjects (7%) had MMSE scores 24 or above (median 29.0, Q_1_ 27.0, Q_3_ 29.8, range 0–30) (Table 1). Two subjects (2%) were reported having dementia (MMSE 0 p and 17 p). Echocardiographic measurements in the whole cohort showed that 144 subjects (68%) had normal LVIDd mm/m^2^, 33 (15%) had mildly abnormal values, 8 (4%) had moderately abnormal values, 16 (8%) had severely high values and 10 (5%) had a dimension under the given reference value. FS was in normal range in 142 subjects (67%), above reference values in 26 (12%) subjects and decreased in 43 subjects (21%). No significant differences were noted between the deceased and the survival group concerning mean LVID size and FS (Table 1).

**Table 1:** Proportions and mean values of background factors, left ventricular measurements and cognitive tests at 68 years and 81 years of age

**Background factor**	**All subjects age 68 (n= 211)**	**Deceased until 81 (n= 88)**	**Survivors to age 81 (n=92)**	***p-value***
Smoking (%) current/former/non	23/57/20	27/54/19	18/61/21	*.483*^*5*^
Alcohol (%) no/rarely/regularly	12/51/37	11/54/35	11/49/40	*.542*^*5*^
ABPI <0.9	24 (11%)	15 (17%)	4 (4%)	*.007*^*4*^
Diabetes mellitus	8 (4%)	5 (6%)	1 (1%)	*.112*^*4*^
Hypertension	120 (57%)	58 (66%)	45 (49%)	*.021*
Angina pectoris	26 (12%)^1^	15 (17%)^1^	6 (7%)	*.026*
Myocardial infarction	22 (10%)^1^	12 (14%)^1^	7 (8%)^1^	*.188*
Stroke	11 (5%)	8 (9%)	3 (3%)	*.127*^*4*^
Education (%) groups	14.5/65/14.5/6	17.6/64.7/12.6/5	10.5/65.3/16.8/7.4	*.093*^*5*^
Hyperlipidemia SBP (mmHg)	10 (5%)^1^	5 (6%)^1^	4 (4%)	*.742*^*4*^
	153.9±20.6	156.5±21.9	151.1±18.1	*.076*
DBP (mmHg)	92.5±10.1	93.2±11.4	91.4±8.7	*.244*
BMI	24.5±3.2^1^	24.7±3.7^1^	24.5±2.5	*.657*
***Echocardiography***				
LVIDd (mm/m^2^)	28.8±4.8	28.2±5.2	29.3±4.3	*.137*
Normotensive	28.8±4.5 (n=91)	28.8±5.4 (n=30)	29.0±3.7 (n=47)	*.853*
Hypertensive	28.8±5.0 (n=120)	27.9±5.1 (n=58)	29.5±4.8 (n=45)	*.105*
LVIDs (mm/m^2^)	19.4±4.4	19.4±4.6	19.4±4.1	*.954*
Normotensive	19.4±4.1 (n=91)	19.5±4.5 (n=30)	19.1±3.5 (n=47)	*.657*
Hypertensive	19.4±4.7 (n=120)	19.3±4.7 (n=58)	19.7±4.8 (n=45)	*.657*
FS (%)	33.1±8.7	31.9±9.2^1^	34.2±8.0	*.072*
Normotensive	32.9±8.9 (n=91)	32.7±9.3 (n=30)	34.2±8.7 (n=47)	*.470*
Hypertensive	33.3±8.6 (n=119)	31.5±9.2 (n=57)	34.3±7.3 (n=45)	*.103*
***Cognitive test, age 68***				
Synonyms	21.0±6.2^2^	20.3±6.0^1^	22.0±6.0^1^	*.069*
Block Design	20.7±6.5^2^	19.6±6.7^1^	22.07±6.0^1^	*.006*
Digit S.	35.0±11.6^1^	32.6±11.1^1^	38.5±11.5	*.001*
Benton VR	5.7±1.7^1^	5.5±1.8^1^	6.0±1.6	*.027*
***Cognitive test, age 81***				
Synonyms			20.0±6.1^3^	
Block Design			14.6±6.5^2^	
Digit S.			28.0±11.2^3^	
Benton VR			4.4±1.8^3^	
MMSE, median			29 (0–30)	

1. Missing data on 1–3 subjects; 2. Missing data on 4–7 subjects; 3. Missing data on > 7 subjects; 4. Fischer’s exact test.5. Kruskal Wallis test. **Abbreviations:** ABPI, ankle brachial pressure index; SBP, systolic blood pressure; DBP, diastolic blood pressure; BMI, body mass index; LVIDd, left ventricular internal dimension at end-diastole in mm/m^2^; LVIDs, left ventricular internal dimension at end-systole in mm/m^2^; FS, fractional shortening. Digit S., Digit Symbol Substitution test; Benton VR, Benton Visual Retention test; MMSE, mini mental state examination.

The subgroups deceased and survivors between baseline and follow-up were compared.

### Ventricular dimensions, cognition and cerebral blood flow

Compared to the subjects with normal LVIDd, subjects with large LVIDd (n=57) had poorer results on the: Synonyms test at baseline (18.8±6.8 vs. 21.7±5.7, *p=0.002*) and on the Digit Symbol Substitution test (32.1±11.2 vs. 35.7±11.8, *p=0.048*). At follow up, surviving subjects with large LVIDd at age 68 (n=26) had poorer results than those with normal LVIDd concerning: Synonyms test (17.1±6.8 vs. 21.2±5.5, *p=0.005*) and Benton Visualisation Retention Test (3.7±1.6 vs. 4.7±1.8, *p= 0.034*). Subjects with low FS performed poorer on Benton Visualisation Retention Test, at baseline, than did subjects with normal FS (5.3±1.5 vs 5.9±1.6, *p=0.033*). No significant differences were noted at follow up.

In a general linear model, in 88 subjects who deceased before age 81, three of the cognitive tests performed at age 68 (Synonyms, Digit symbol and Benton Visual Retention test) were negatively associated with LVIDd mm/m^2^ (Table 2). In 92 survivors at age 81, two of the cognitive tests performed at age 81 (Synonyms and Benton Visual Retention test) were negatively associated with LVIDd mm/m^2^ (Table 2). In both models, regression analysis was adjusted for education, antihypertensive therapy and systolic blood pressure in the same year as cognitive assessment. No associations were noted between FS and cognitive tests (data not shown).

**Table 2:** **Association between left ventricular internal dimension in diastole (LVIDd mm/m**^**2**^**), as a dependent variable, and each of the cognitive tests at age 68, estimated for subjects deceased before age 81, and each of the cognitive tests at age 81 for the survivors, in an adjusted linear model**

	**LVIDd mm/m**^**2**^				
	**B**	**t**	**S.E.**	***df***	***p***
**Cognition at age 68 in deceased before age 81**^**(1)**^**;*n=88***					
Synonyms	-.322	.111	−2.891	112	.005**
Block Design	-.224	.121	−1.856	116	*.066*
Digit Symbol	-.461	.196	−2.353	115	.020*
Benton Visual Retention	-.090	.030	−2.955	115	.004**
**Cognition at age 81**^**(2)**^**;*n=92***					
Synonyms	-.372	.140	−2.651	84	.010*
Block Design	-.187	.163	−1.148	88	*.255*
Digit Symbol	-.540	.301	−1.795	80	*.077*
Benton Visual Retention	-.107	.050	−2.148	75	.035*

**(1)** adjusted for education, antihypertensive medication at age 68, and systolic blood pressure at age 68.

**(2)** adjusted for education, antihypertensive medication at age 81, and systolic blood pressure at age 81.

When analyzing longitudinal impact of cardiac function on cerebral blood flow, we found in a general linear model no association between CBF and FS in a whole cohort. After stratification according to the presence of hypertension at age 68, rCBF in left frontal, both temporal, right parietal, occipital areas and right basal nuclei, were all associated with FS in the normotensive subgroup, also after adjusting for systolic blood pressure at age 81, active smoking at age 68, alcohol consumption at age 68 and stroke (Table 3). No associations were found between rCBF and FS in hypertensives at age 68, nor between rCBF and LVIDd.

**Table 3:** Association between regional Cerebral Blood Flow (rCBF) at age 81, as a dependent variable, and Fractional Shortening (FS) at age 68 in normotensive (n=37) and hypertensive subjects (n=35) at age 68 in an adjusted linear model

	**Normotensive**		**Hypertensive**	
**rCBF**	**B (S.E.)**^**(1)**^	***p***	**B (S.E.)**^**(1)**^	***p***
**Frontal right**	.225 (.129)	*.091*	-.147 (.151)	*.361*
**Frontal left**	.292 (.130)	.033*	-.141 (.145)	*.340*
**Temporal r**	.201 (0.78)	.016*	-.167 (.129)	*.207*
**Temporal l**	.237 (.102)	.027*	-.088 (.130)	*.201*
**Parietal r**	.257 (.124)	.047*	-.159 (.160)	*.330*
**Parietal l**	.242 (.141)	*.096*	-.143 (.171)	*.409*
**Occipital**	.357 (.141)	.017*	-.172 (.151)	*.263*
**Basal nuclei r**	.325 (.135)	.023*	-.082 (.188)	*.669*
**Basal nuclei l**	.238 (.137)	*.093*	-.009 (.193)	*.964*

^**(1)**^ Adjusted for: systolic blood pressure at age 81, active smoking at age 68, alcohol consumption at age 68 and stroke.

### Hypertensive vs. normotensive subgroup

Comparison of the mean dimensions of the left cardiac ventricle and FS between normotensive and hypertensive subjects showed no significant differences, neither in the deceased, nor in survivors. Furthermore the results of the cognitive tests did not differ with respect to the blood pressure status. At age 68, 91 of the study subjects (56%) were normotensive, whereas 71 subjects (44%) were hypertensive. Subjects with antihypertensive medication were excluded in this analysis (n=49). Between baseline and follow-up, 48% of the hypertensive subjects deceased whereas corresponding proportion for normotensive subjects was 33% (p=0.050). At age 81, 60 subjects (92%) were normotensive and 5 subjects (8%) were hypertensive. 38 subjects (58%) were normotensive at both examinations, at age 68 as well as at age 81, 2 subjects (3%) were normotensive at age 68 and hypertensive at age 81, 3 subjects (5%) were hypertensive at both examinations and 22 subjects (34%) were hypertensive at age 68 and normotensive at age 81. 88% of the surviving hypertensives at age 68 became normotensive by the follow-up whereas 95% of the surviving normotensive 68-year-old men remained normotensive at follow-up.

### Drop out analysis

Background data for the participants versus excluded subjects are presented in Table 4. Excluded subjects had significantly higher BMI, lower scores on the Synonyms Test and the Block Design test. No other differences were found between the groups.

**Table 4:** Proportion and mean values of background factors and cognitive tests

**Background factors**	**Participants (n= 211)**	**Excluded (n=289)**	***p-value***
Smoking (%) current /former/non	23/57/20	22/51/26	*.121*
Alcohol (%) no/rarely/regularly	12/51/37	13/51/36^3^	*.608*
ABPI <0.9	24 (11%)	39 (14%)	*.480*
Diabetes mellitus	8 (4%)	17 (6%)	*.289*
Hypertension	120 (57%)	157 (54%)^3^	*.961*
Angina pectoris	26 (12%)^1^	38 (13%)^1^	*.778*
Myocardial infarction	22 (11%)^1^	19 (7%)^1^	*.122*
Stroke	11 (5%)	18 (6%)	*.632*
Education (%), groups	14.5/65/14.5/6	15/70/12/3	*.202*
Hyperlipidaemia	10 (5%)^1^	13 (5%)^3^	*.993*
SBP (mmHg)	153.9±20.6	153.2±22.9^3^	*.737*
DBP (mmHg)	92.5±10.1	92.7±11.7^3^	*.848*
BMI	24.5±3.2^1^	25.5±3.3^4^	*.001*
**Cognitive test, age 68**			
Synonyms	21.0±6.2^2^	19.6±6.3^4^	*.021*
Block design	20.7±6.5^2^	18.9±6.3^4^	*.002*
Digit Symbol	35.0±11.6^1^	33.5±11.8^4^	*.170*
Benton VR	5.7±1.7^1^	5.4±1.7^4^	*.051*

^1^Missing data on 1–3 subjects; ^2^ Missing data on 4–7 subjects; ^3^ Missing data on 8–17 subjects; ^4^ Missing data on 18–30 subjects; ^5^ Missing data on 31–36 subjects.

**Abbreviations:** ABPI, ankle brachial pressure index; SBP, systolic blood pressure; DBP, diastolic blood pressure; BMI, body mass index. Digit S., Digit Symbol Substitution test; Benton VR, Benton Visual Retention test.

The study participants and excluded subjects (without and incomplete echocardiographic examination) were compared.

## Discussion

In this male, general population cohort study, we found that subjects with enlarged LVIDd at age 68 had poorer results on verbal and speed-performance tests at baseline and verbal and visuo-spatial tests 14 years later on, compared to the subjects with normal LVIDd. Low FS was associated with decreased results on visuo-spatial tests at baseline examination. In an adjusted linear model, we observed an association between larger LVIDd and poorer results during both cognitive testing surveys: at age 68 for those who deceased before age 81, and at age 81 for the survivors. In normotensives, in an adjusted linear model, lower FS predicted decreased CBF in a majority of brain areas.

### Blood pressure, cognition and cerebral blood flow

Several studies have reported that hypertension, both systolic and diastolic blood pressure (BP), play a key role in cognitive impairment and has an impact on CBF over time. Memory, attention and abstract reasoning have been reported as particularly vulnerable to hypertension [28]. The recommended guidelines for treatment of hypertension in this cohort during the eighties and nineties were >160/90 mm Hg, which might indicate that the hypertensive group represented individuals with more deranged blood pressure and higher atherosclerotic risk, compared to later studies. There was a slithly higher proportion of hypertensive subjects without antihypertensive medication at baseline that deceased before follow-up than normotensive subjects (48% vs. 33; p=0.050). In the analysis of changes of BP, we found that 95% of the surviving normotensive subjects at age 68 remained normotensive at age 81, whereas 88% of surviving hypertensive subjects at age 68 became normotensive at age 81. Hypertension in midlife most likely negatively affects cognition and contributes to dementia later in life whereas low BP, especially DBP, in older adults is associated with increased risk for dementia [29]. Another explanation on the relationship between hypertension and rCBF could be the fact that cerebral autoregulation compensates for variations in mean arterial blood pressure within a certain range, however both age [30] and chronic hypertension may lead to CBF reductions [31].

### Echocardiography and cognition

It has been reported that an increase in left ventricular internal dimension is a risk factor for congestive heart failure in persons not having had myocardial infarction [8]. Furthermore, large LVIDd has been reported as an independent predictor for all-cause mortality or hospitalization for cardiac causes [32]. We have in an earlier study found that ambulatory ST segment depression, as a measure of ischemic heart disease (IHD), was associated with lower cognitive function, both visuospatial and verbal [14]. In our material, 144 subjects (68%), had a normal LVIDd, whereas 57 (27%) had enlarged. Subjects with enlarged LVIDd at age 68 had poorer results on verbal and speed-performance tests at baseline and verbal and visuo-spatial tests 14 years later on, compared to subjects with normal LVIDd. Low FS was associated with decreased results on visuo-spatial tests at baseline. The Synonyms test was negatively associated to LVIDd both at baseline and at follow-up. The Synonyms test examine verbal ability, a measure of crystallized intelligence, which is regarded as being more age stable than fluid intelligence (speed performance functions) [33]. Function of semantic memory is connected to activation of the left part of the frontal lobe and to the left part of the temporal lobe [34]. These areas are in conjunction with affected areas in the CBF examination (see below). Impairment of semantic memory has been associated with Alzheimer’s disease [35]. Visuo-spatial ability was negatively associated with LVIDd both at baseline as well as at follow-up. Our findings are supported by a previous study, where LVM was found to be negatively associated with visuo-spatial and verbal memory [6]. Contrary to a previous report [5], we didn’t find any correlations at follow-up between MMSE and left ventricular measurements. One possible explanation is that 94% of the participants at follow-up had MMSE results above 24 points and the different parts of the MMSE test seem to be unequal in identifying subjects with mild cognitive disturbances [36]. Similar findings have been reported by an earlier study where no associations were found between MMSE, DBP and SBP [37].

### Echocardiography and cerebral blood flow

In the study of of Sierra et al. [7], the presence of LVH in middle-aged patients with essential hypertension was associated with a reduction of rCBF in the striatum area. We found previously an association between ST-segment depression (STDE), as a measure of ischemic heart disease (IHD), and reduced rCBF in frontal, temporal and parietal regions [38]. Since hypertension is such a great risk factor for cardiovascular disease [11], we decided to stratify study subjects into hypertensive and normotensive subjects when examining the associations between LVIDd, FS and rCBF. Inverse associations were found between FS and rCBF only in normotensive subjects. A possible explanation for this finding may be that subjects with early hypertension could have already developed changes in cerebral vasculature which erase the effect of cardiac changes on CBF in the senescence. Another explanation could be a selective mortality of those subjects with highest vascular load, early including hypertension, and survival of men with protective factors which enabled longevity. The absence of an association between LVIDd and rCBF may be explained by the fact that FS better mirrors the contractility of the heart and cardiac output, and therefore interact closer with rCBF than do LVIDd. In the study of Loncar et al. ejection fraction was found to be an independent determinant of impaired CBF in patients with heart failure although other studies have not showed no such correlation [39]. However, only about half of heart failure patients have deranged ejection fraction [12]. The underlying mechanisms between heart failure and cognitive impairment are yet not fully known. One might speculate whether a low FS and decreased cardiac output account for cerebral hypoperfusion or an advanced atherosclerosis with deranged cerebral vascular function, or perhaps a combination of both.

### Limitations

Several limitations in our study merit to be commentated. Interpretation of our study is limited to men. Women might have a better autoregulation than men, according to the study of Deegan et. al [40]. Furthermore, women with mild hypertension showed greater reductions in BP, as they age, and have an increased risk of developing heart failure [41]. We are partly dealing with the effects of selection bias concerning subjects examined with CBF measurement at the age of 83, because subjects with severe vascular risk profiles might not have reached such high age. Therefore, observed associations might have been even stronger among the deceased men. However, in this group with healthy and cognitively intact elderly men, we still manage to find an association between FS and CBF. Another limitation is the definition of hypertension (>160 mmHg systolic, >90 mmHg diastolic or being on antihypertensive medication), which accept higher limits and leading to the fact that we have subjects with mild hypertension classified in the study as normotensive subjects. M-mode echocardiography is an old and widely used method, although it’s role has become less important nowadays compared to when this study was conducted. M-mode measurements are valid only when LV geometry is normal and may be misleading in case of remodeling. There is a variability in examining patients with M-mode echocardiography and also concerning measurements after examination. As a strength of this study we should express that measurements were made by two experienced technicians from the same laboratory using the same standards and equipment. Most studies examining the association between left cardiac ventricular function and cognition, use LVM as cardiac variable. LVM progressively increases with age and in the presence of risk factors like hypertension. Increased LVM may be due to different patterns of remodeling. FS reflects partly the contractility of the left cardiac ventricle and, for individuals with regional wall abnormalities, FS is only specific for the base of the heart.

## Conclusions

In this population-based sample of elderly men, we found that increased left ventricular internal dimension in diastole, at age 68, is associated with decreased verbal and spatial ability, both at baseline as well as at follow-up. Diminished fractional shortening in normotensive 68-year-old men predicts future cerebral perfusion deficits. In order to find new ways of identifying subjects at risk, and preventing cognitive decline in elderly, more knowledge about the mechanisms between cardiovascular disease and cognition is needed.

## Abbreviations

ABPI: Ankle brachial pressure index; BP: Blood pressure; BSA: Body surface area; CBF: Cerebral blood flow; DBP: Diastolic blood pressure; EF: Ejection fraction; FS: Fractional shortening; IHD: Ischemic heart disease; LV: Left ventricle; LVH: Left ventricular hypertrophy; LVM: Left ventricular mass; LVIDd: Left ventricular internal dimension in diastole; LVIDs: Left internal ventricular dimension in systole; MABP: Mean arterial blood pressure; rCBF: Regional cerebral blood flow; ROI: Region of interest; SBP: Systolic blood pressure; STDE: ST segment depression.

## Competing interests

The authors declare that they have no competing interests.

## Authors’ contributions

Organization of cerebral blood flow measurements was carried out by PW. Planning of the study was carried out by LF, PW, AS and SE. Preliminary analysis of the data was carried out by LF, AS and SE jointly. The first draft of the paper was written by LF. All authors contributed to revisions and to the final version of the manuscript. All authors read and approved the final manuscript.

## Pre-publication history

The pre-publication history for this paper can be accessed here:

http://www.biomedcentral.com/1471-2318/13/45/prepub
